# 

**Published:** 1852-08

**Authors:** 


VOL. I. NEW SERIES. No. 4.

Medical Times

### THE NORTH-WESTERN MEDICAL AND SURGICAL JOURNAL.

PUBLISHED MONTHLY, AT TWO DOLLARS PER ANNUM.

EDITED BY

W. B. HERRICK, M.D.,

PROFESSOR OF ANATOMY AND PHYSIOLOGY IN RUSH MEDICAL COLLEGE; SURGEON TO THE U.S. MARINE HOSPITAL, AND ONE OF THE SURGEONS OF THE ILLINOIS GENERAL HOSPITAL, ETC.

ASSISTED BY

H. A. JOHNSON, M.D.

---

**AUGUST, 1852.**

---

CHICAGO:

BALLANTYNE & CO., PRINTERS, PUBLISHERS, & BOOKBINDERS

NEW POST-OFFICE BUILDINGS, COR. OF CLARK AND RANDOLPH-STS.,

OPPOSITE THE SHERMAN HOUSE.

1852.

VOL. IX. WHOLE SERIES. No. 4.

